# Supplementary material for: Bioinspired ruthenium-manganese-oxygen complex for biocatalytic and radiosensitization therapies to eradicate primary and metastatic tumors
Source: Nat Commun. 2025 Aug 16;16:7640. doi: 10.1038/s41467-025-62999-x (PMC12357870; doi:10.1038/s41467-025-62999-x)
Supplement: Supplementary file 1 — Supplementary Information [file 41467_2025_62999_MOESM1_ESM.pdf]

Supplementary Information

Bioinspired ruthenium-manganese-oxygen complex for biocatalytic and radiosensitization therapies to eradicate primary and metastatic tumors

This PDF file includes:

Supplementary Figures

Supplementary Tables

Supplementary Methods

Supplementary References

Supplementary Figures

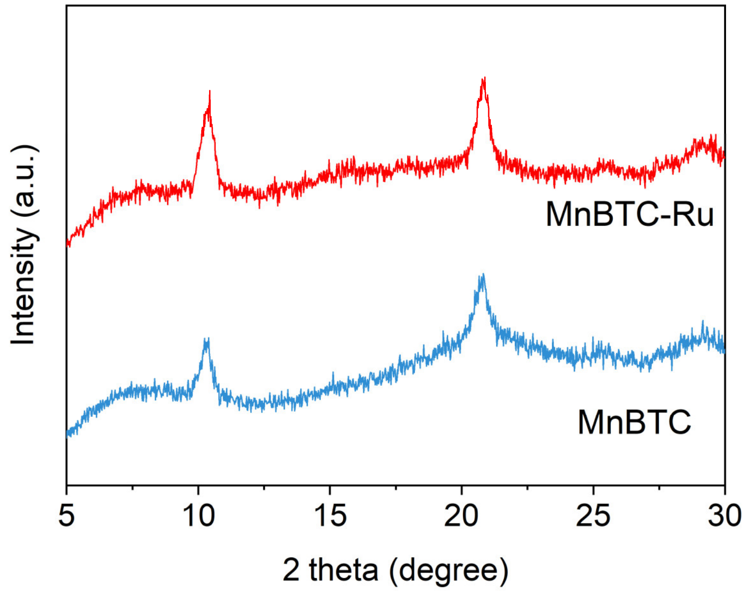

Supplementary Fig. 1. X-ray diffraction patterns of MnBTC-Ru and MnBTC. a.u. indicates the arbitrary units. Source data are provided as a Source Data file.

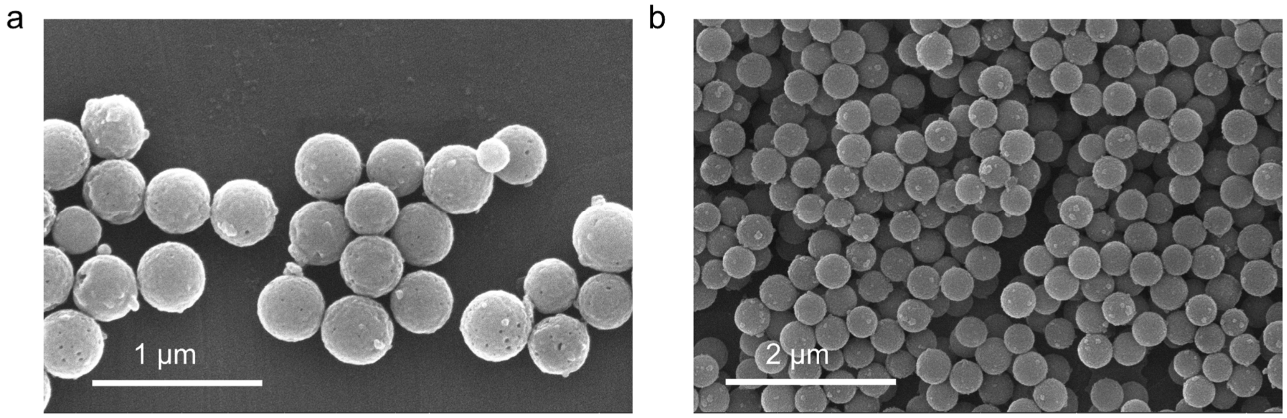

Supplementary Fig. 2. Scanning electron microscope (SEM) images of **a** MnBTC and **b** MnBTC-Ru.

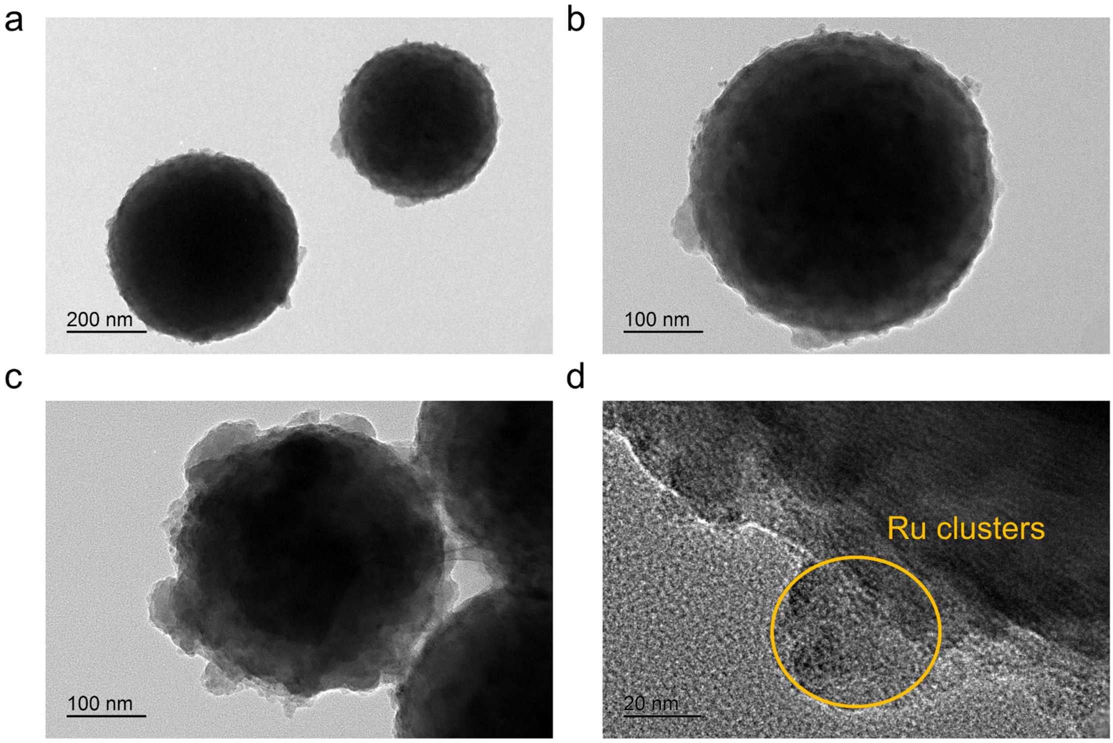

Supplementary Fig. 3. Transmission electron microscopy (TEM) images of MnBTC-Ru at varying magnifications: **a** 200 nm, **b** 100nm, **c** 100 nm, **d** 20 nm.

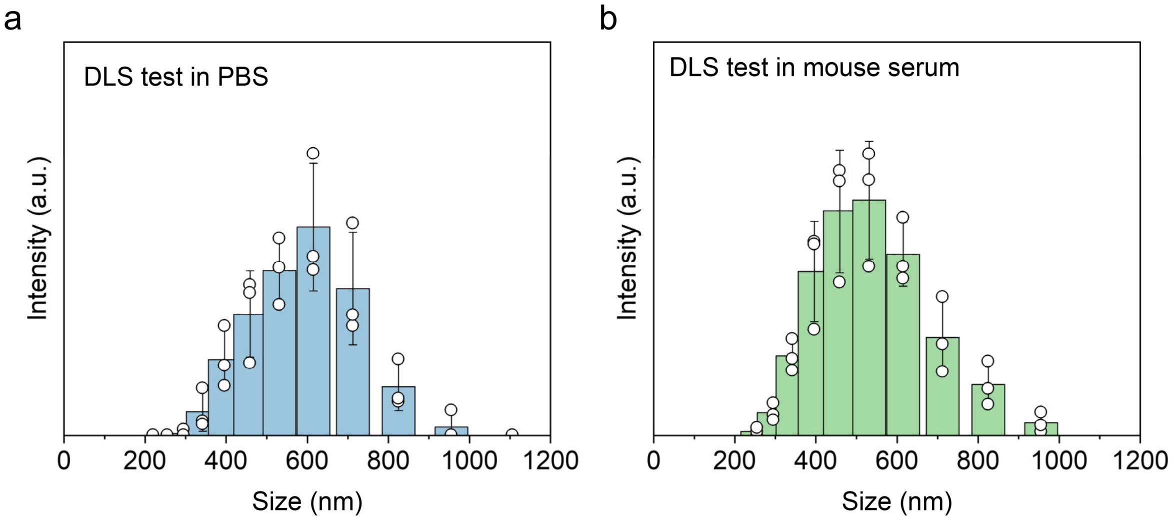

Supplementary Fig. 4. Dynamic light scattering (DLS) analysis of MnBTC-Ru conducted in **a** phosphate-buffered saline (PBS) and **b** mouse serum (n = 3 independent experiments, data are presented as mean ± SD). a.u. indicates the arbitrary units. Source data are provided as a Source Data file.

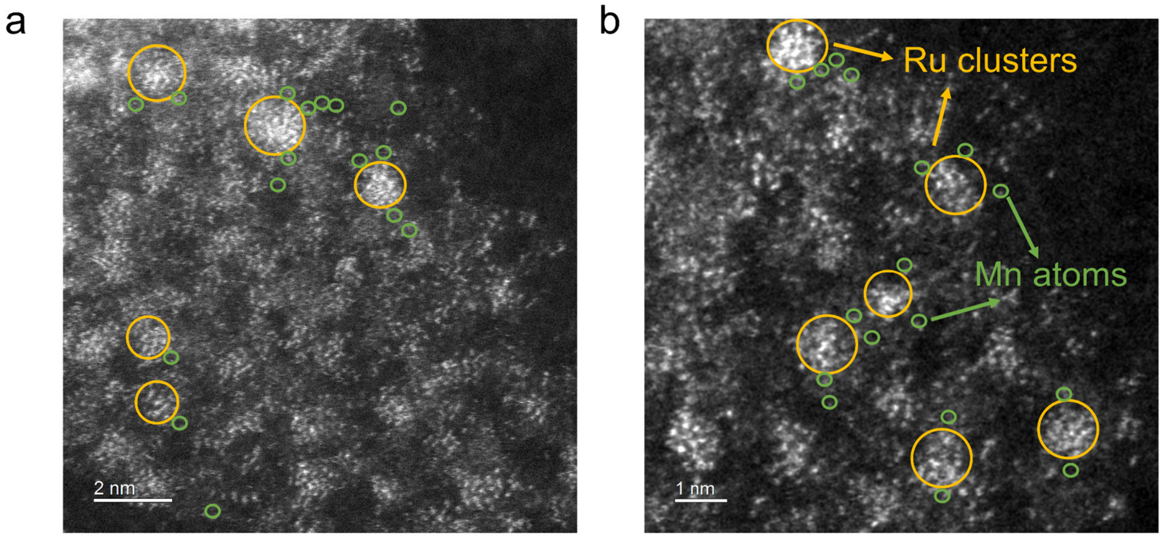

Supplementary Fig. 5. Atomic-resolution high-angle annular dark-field scanning transmission electron microscopy (HAADF-STEM) images of MnBTC-Ru at different magnifications: **a** 2 nm, **b** 1 nm.

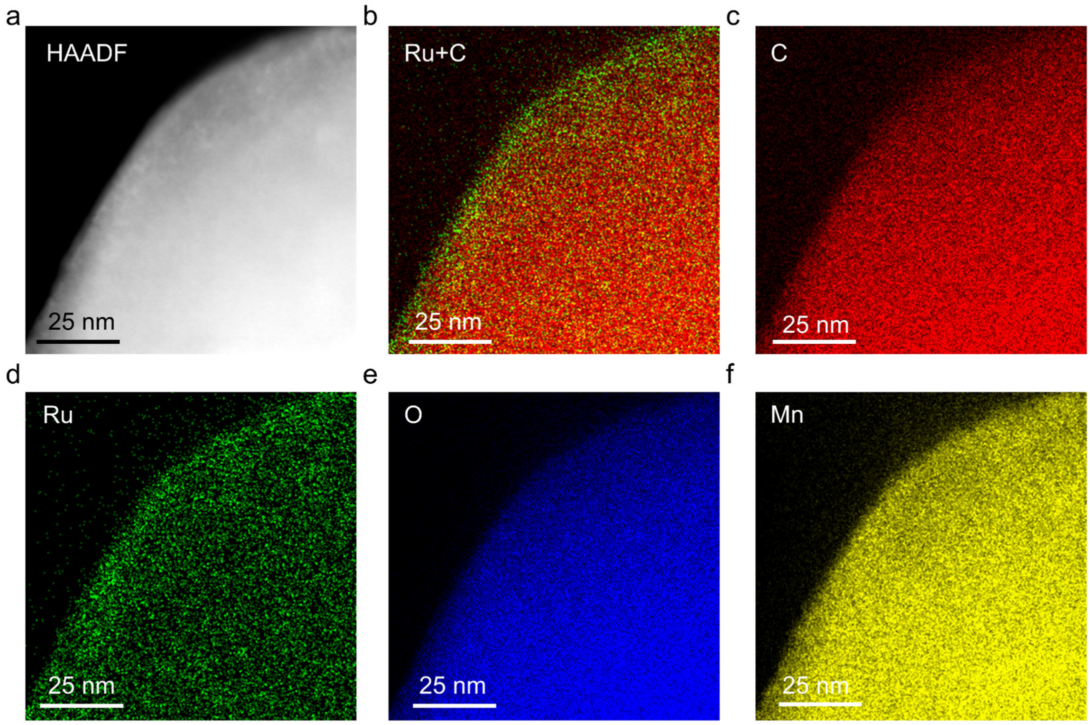

Supplementary Fig. 6. a HAADF-STEM image and **b-f** corresponding energy-dispersive spectroscopy (EDS) mapping of MnBTC-Ru.

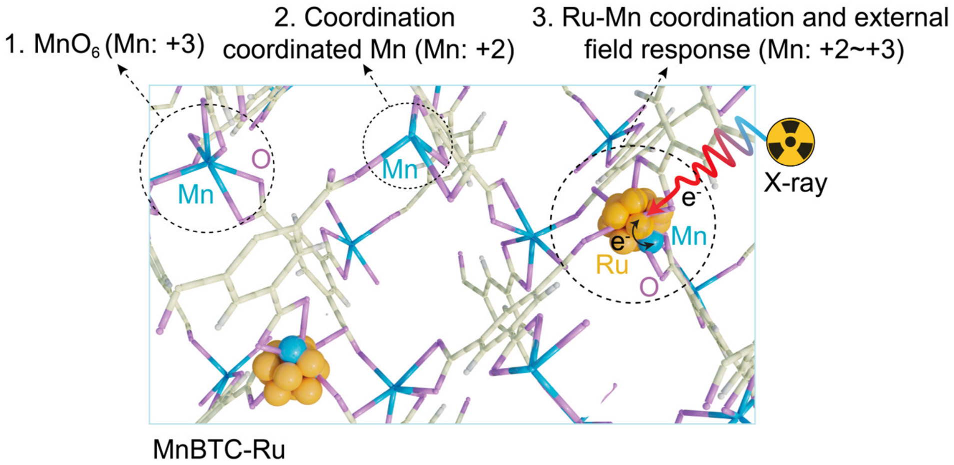

Supplementary Fig. 7. Schematic illustration of the valence states of Mn under various coordination environments in MnBTC-Ru.

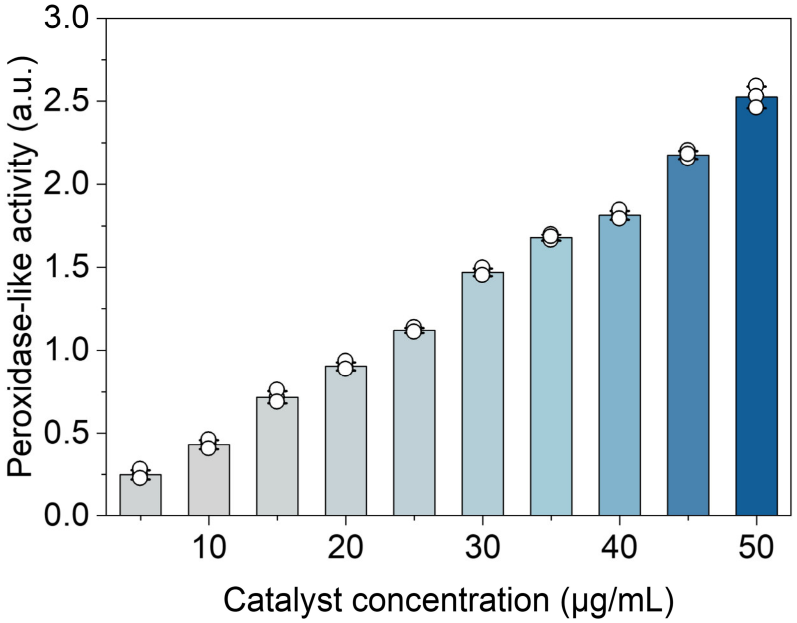

Supplementary Fig. 8. Concentration-dependent peroxidase-mimetic activity of MnBTC-Ru (n = 3 independent experiments, data are presented as mean ± SD). a.u. indicates the arbitrary units. Source data are provided as a Source Data file.

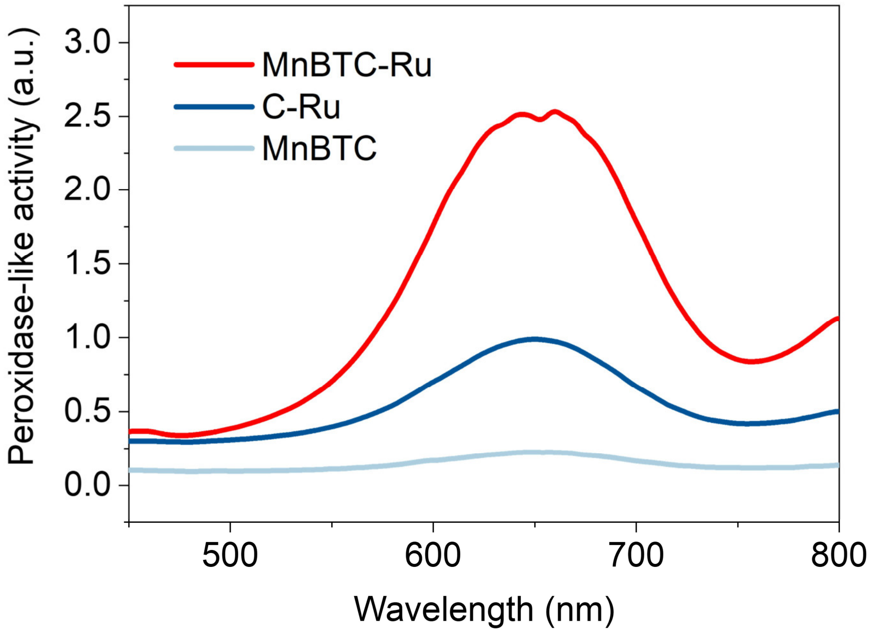

Supplementary Fig. 9. The increase in absorbance of 3,3',5,5'-tetramethylbenzidine (TMB) at 652 nm indicated peroxidase-like activity of MnBTC-Ru. a.u. indicates the arbitrary units. Source data are provided as a Source Data file.

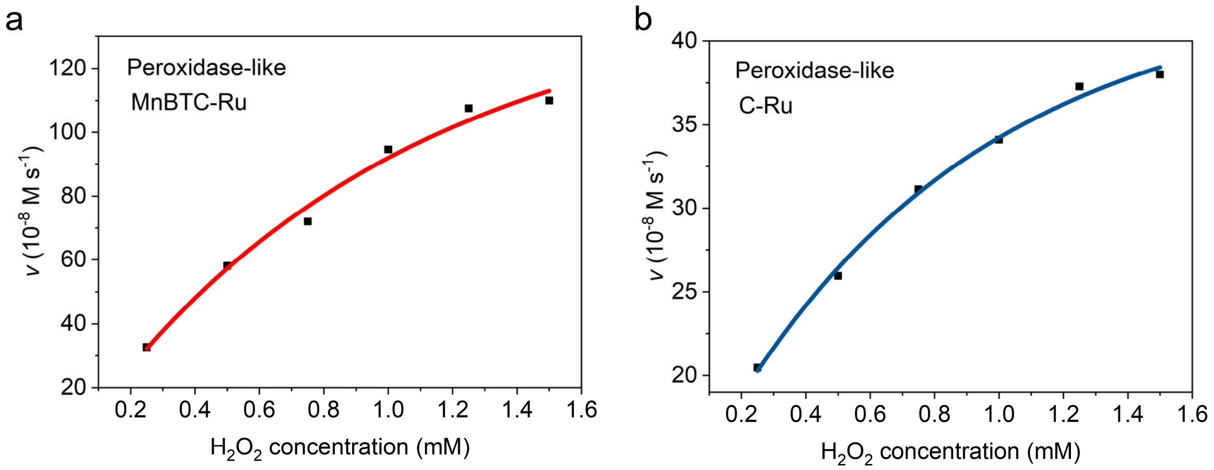

Supplementary Fig. 10. Michaelis-Menten kinetic analysis for **a** MnBTC-Ru and **b** C-Ru with hydrogen peroxide (H_2O_2) as substrate. *v* indicates the initial reaction rates. Source data are provided as a Source Data file.

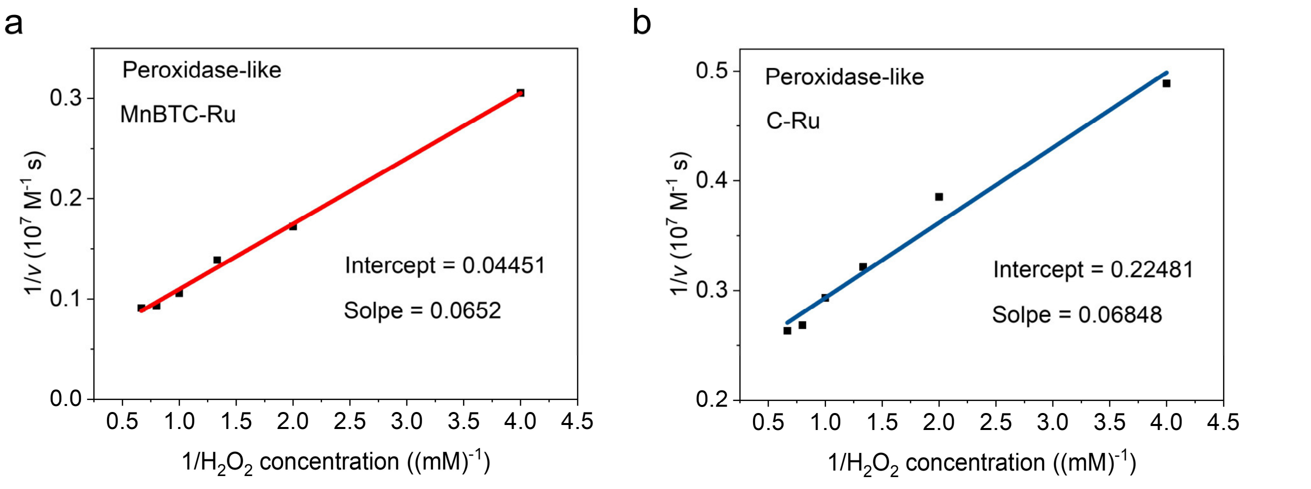

Supplementary Fig. 11. Lineweaver–Burk plot for **a** MnBTC-Ru and **b** C-Ru with H_2O_2 as substrate. Source data are provided as a Source Data file.

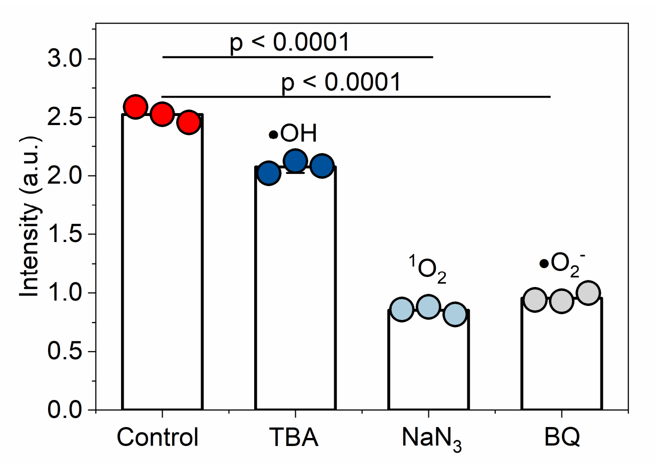

Supplementary Fig. 12. Tert-butanol (TBA) to quench •OH, benzoquinone (BQ) to quench $\bullet O_2^-$, and sodium azide (NaN_3) to quench $^1O_2$ during the biocatalytic process of TMB ($n = 3$ independent replicates, data are presented as mean ± SD). Statistical significance was assessed using the one-way ANOVA for multiple-group comparisons, followed by Tukey's two-tailed post-hoc test for pairwise analysis, all tests were two-sided. a.u. indicates the arbitrary units. Source data are provided as a Source Data file.

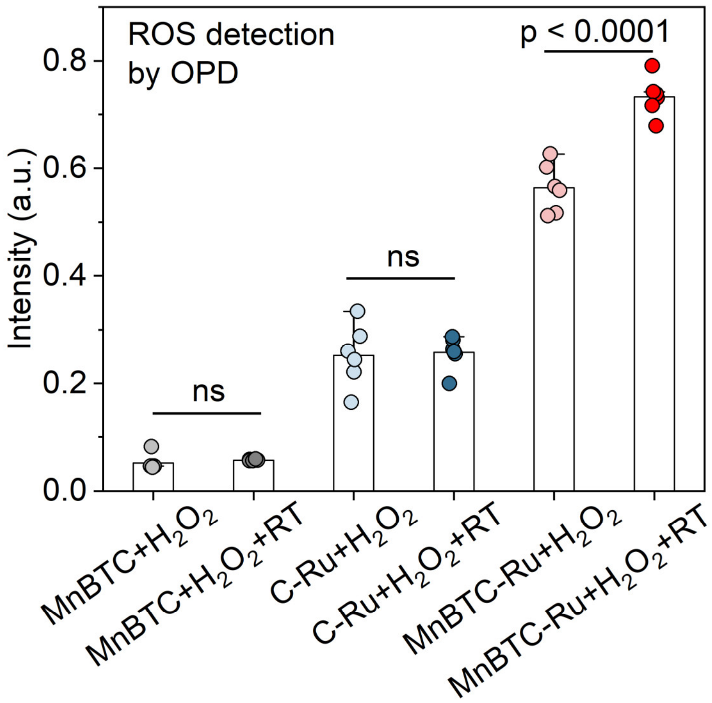

Supplementary Fig. 13. The proportion of reactive oxygen species (ROS) produced by each component is detected by o-phenylenediamine (OPD) (n = 6 independent experiments, data are presented as mean ± SD). Statistical significance was assessed was calculated using two-tailed Student's t-test, all tests were two-sided. a.u. indicates the arbitrary units. RT indicates radiotherapy. ns indicates no significance. Source data are provided as a Source Data file.

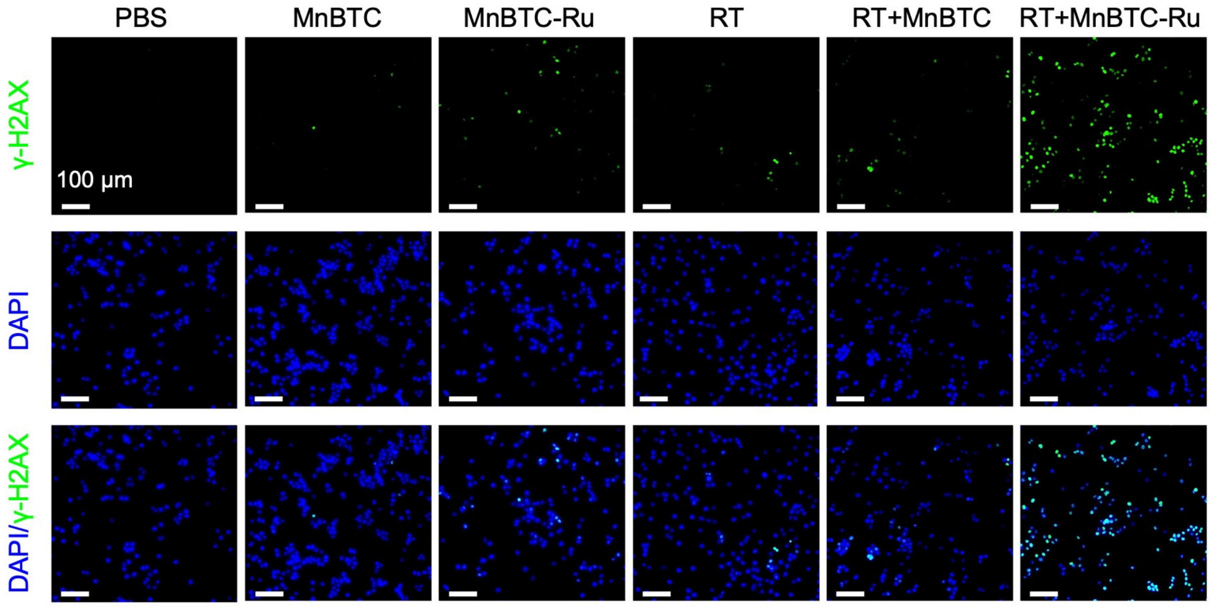

Supplementary Fig. 14. γ-H2AX immunofluorescence assay. Scale bar = 100 μm. Experiments were repeated independently three times with similar results. γ-H2AX indicates phosphorylated histone H2A.X at Ser139, DAPI indicates 4′,6-diamidino-2-phenylindole.

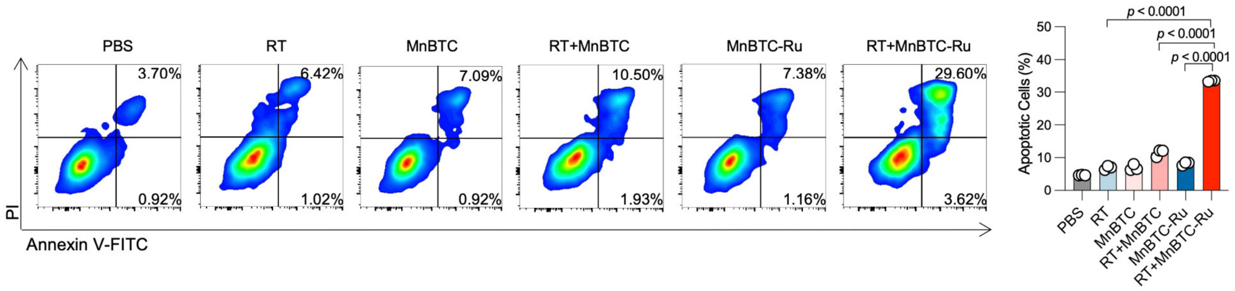

Supplementary Fig. 15. The Annexin V/PI analysis of CT26 cells in different groups. The graph showed the percentage of apoptotic cells (early apoptotic, late apoptotic) in different groups (n = 3 independent replicates, data are presented as mean ± SD). Statistical significance was assessed using the one-way ANOVA for multiple-group comparisons, followed by Tukey's two-tailed post-hoc test for pairwise analysis, all tests were two-sided. PI indicates propidium iodide, FITC indicates fluorescein isothiocyanate. Source data are provided as a Source Data file.

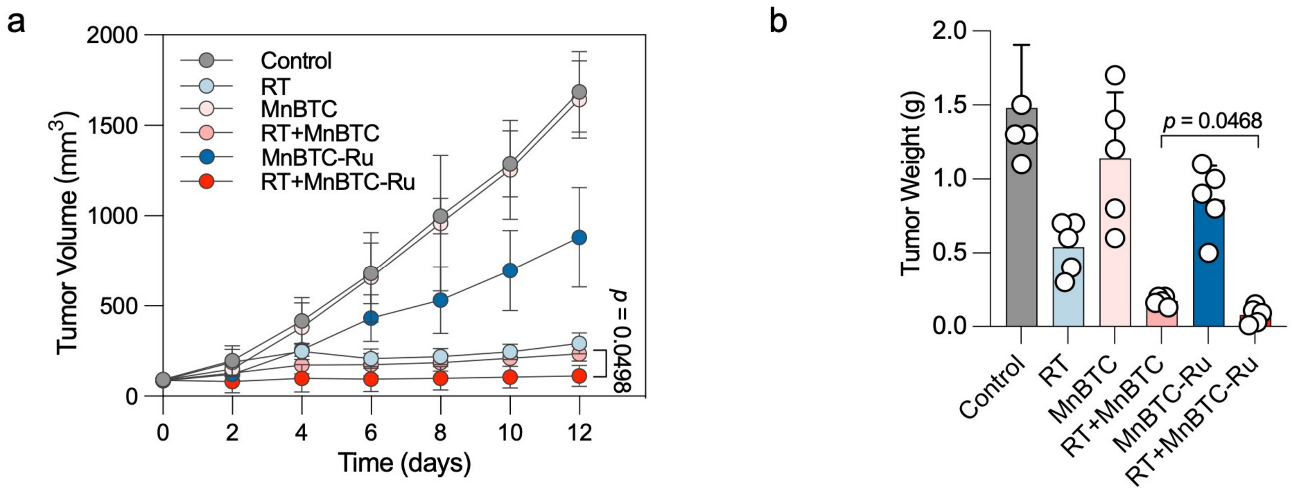

Supplementary Fig. 16. a Average tumor growth curves and **b** tumor weight of CT26 tumor-bearing mice after different treatments ($n = 5$ independent replicates, data are presented as mean ± SD). Statistical significance was assessed using the one-way ANOVA for multiple-group comparisons, followed by Tukey's two-tailed post-hoc test for pairwise analysis, all tests were two-sided. Source data are provided as a Source Data file.

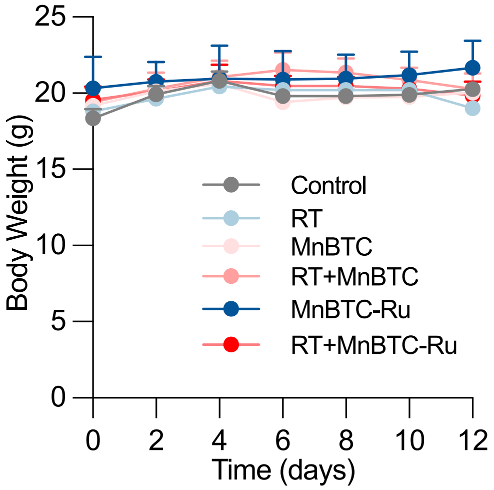

Supplementary Fig. 17. Body weight of CT26 tumor-bearing mice during different treatments ($n = 5$ independent replicates, data are presented as mean ± SD). Source data are provided as a Source Data file.

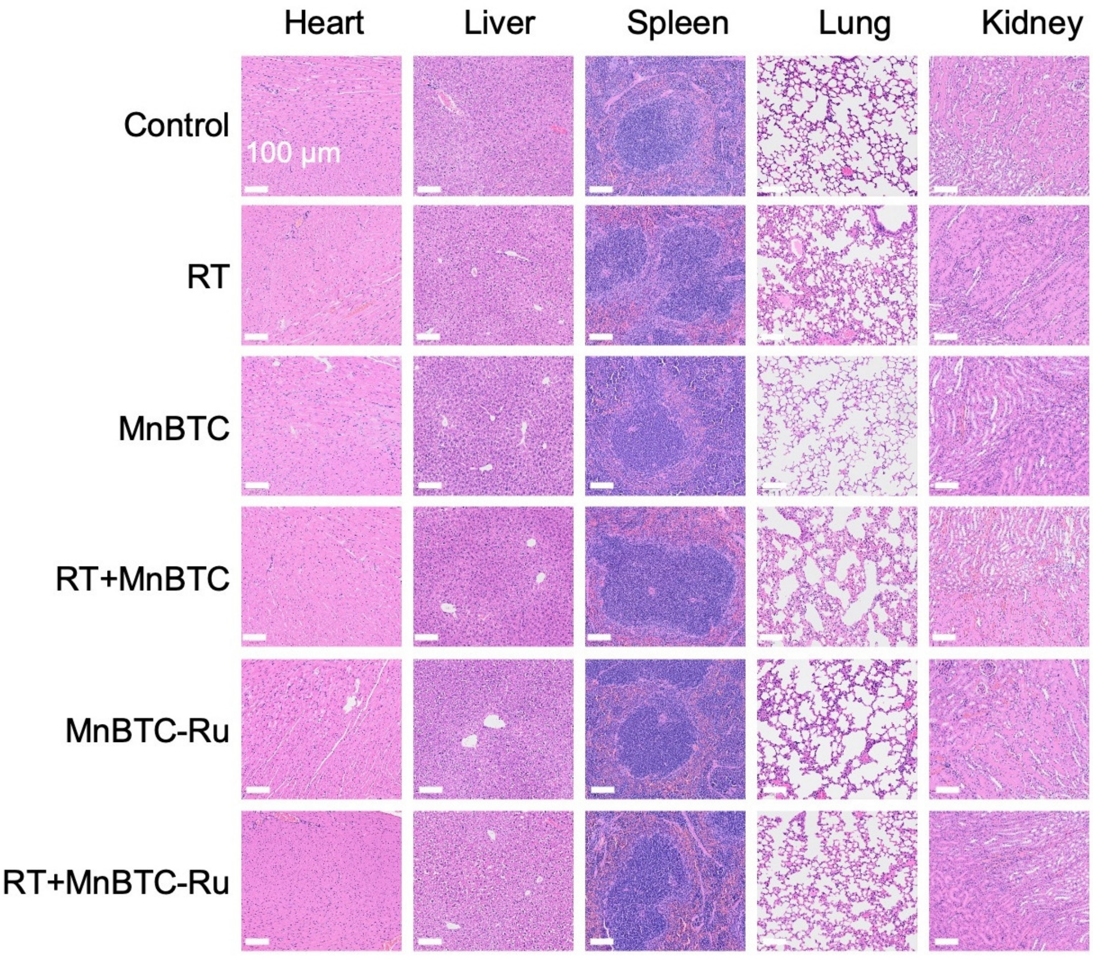

Supplementary Fig. 18. Representative H&E staining of major organs from CT26 tumor-bearing mice after different treatments (scale bar = 100 μm), experiments were repeated independently three times with similar results. H&E indicates hematoxylin and eosin.

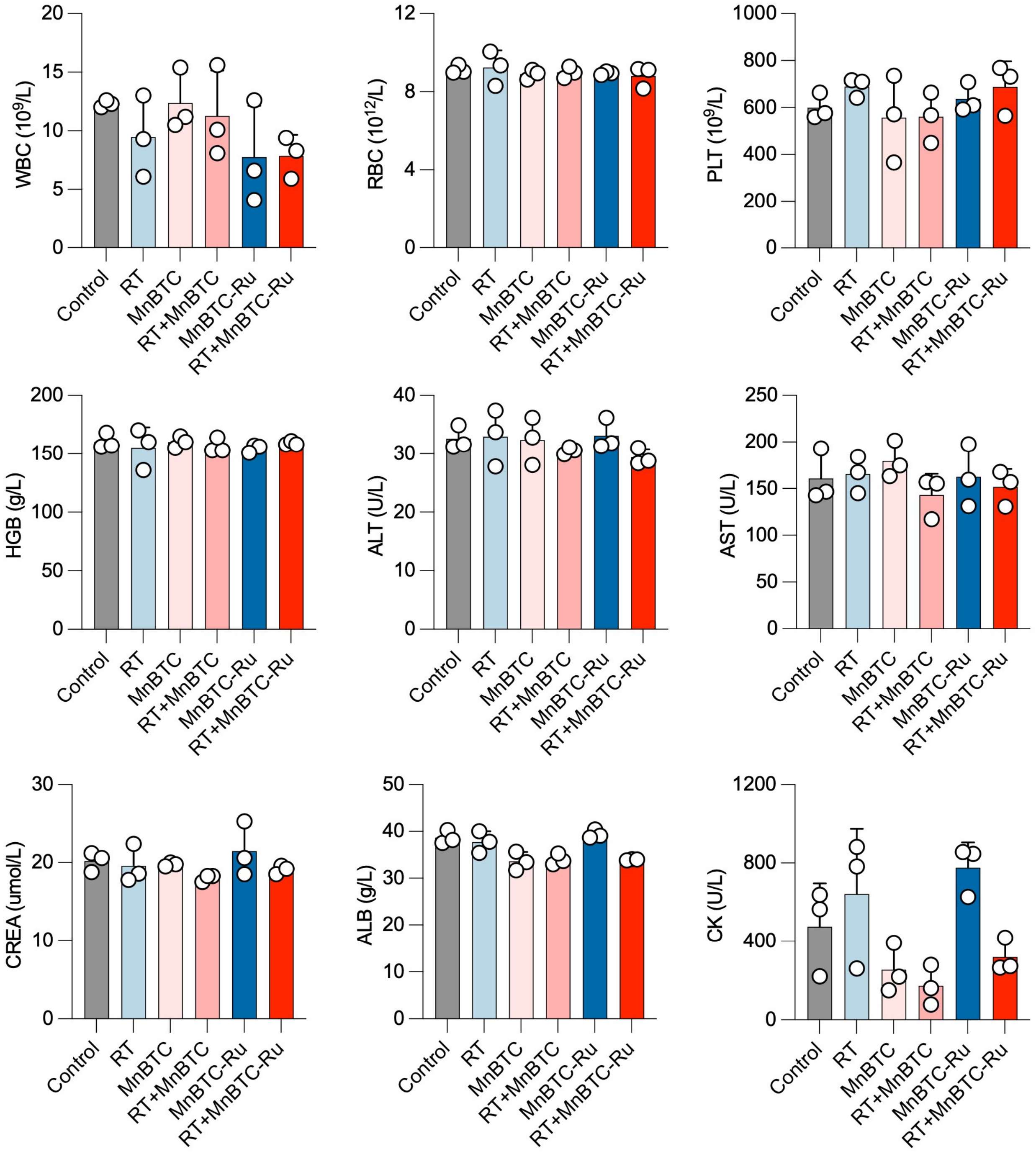

Supplementary Fig. 19. Results of blood chemistry parameters from mice after different treatments (n = 3 independent replicates, data are presented as mean ± SD). Blood chemistry parameters include white blood cell (WBC), red blood cell (RBC), platelet count (PLT), hemoglobin (HGB), alanine aminotransferase (ALT), aspartate aminotransferase (AST), creatinine (CREA), albumin (ALB), and

Creatine Kinase (CK). Source data are provided as a Source Data file.

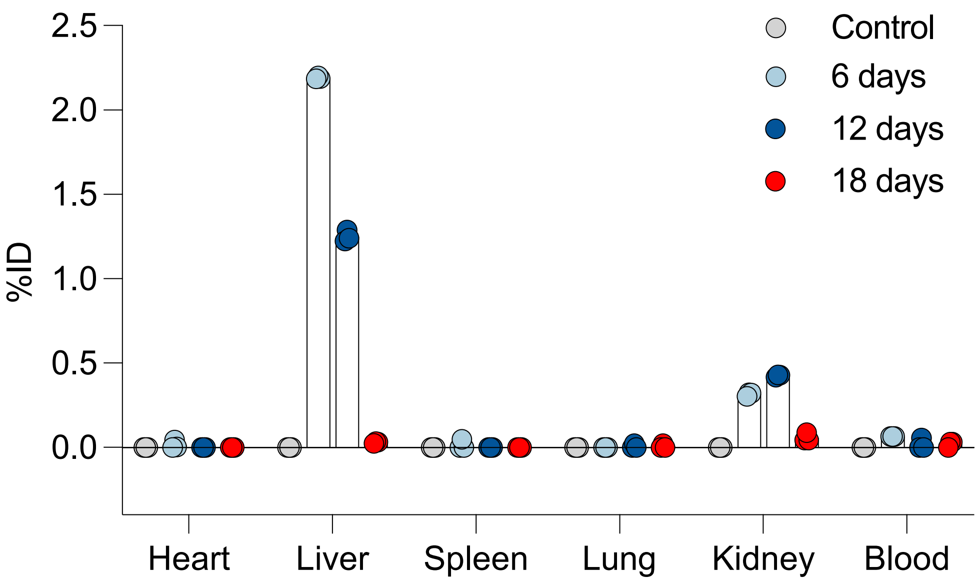

Supplementary Fig. 20. Biodistribution of MnBTC-Ru in major organs and blood of mice at different time points after intratumoral injection of MnBTC-Ru (n = 3 independent replicates, data are presented as mean ± SD). %ID indicates the percentage of injected dose. Source data are provided as a Source Data file.

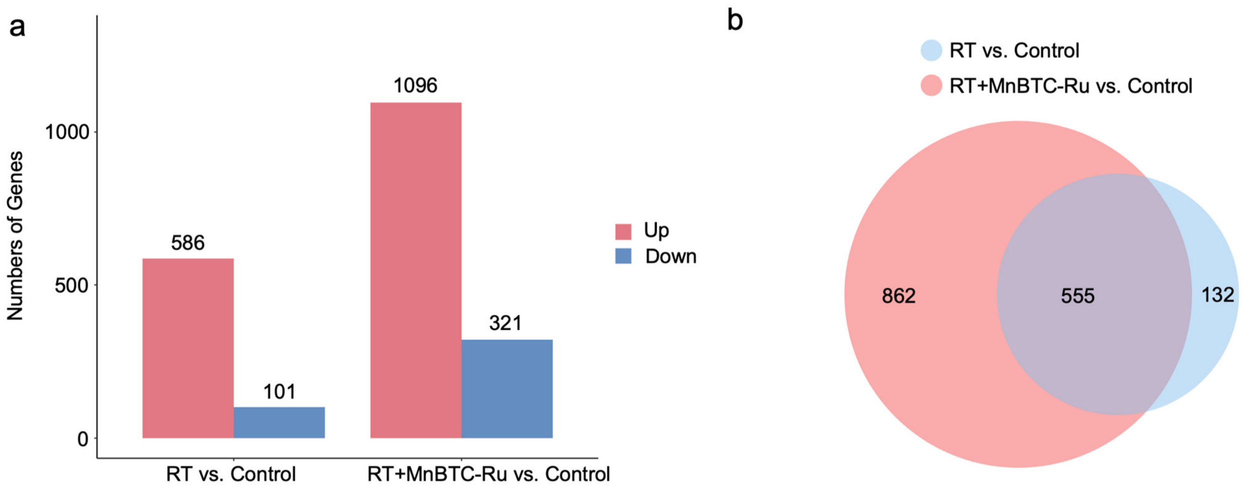

Supplementary Fig. 21. Differentially expressed genes in different groups. **a** Bar charts and **b** Venn diagrams depict the differentially expressed genes identified through tumor RNA sequencing among three

groups: the Control group, the RT group, and the RT+MnBTC-Ru group. Source data are provided as a Source Data file.

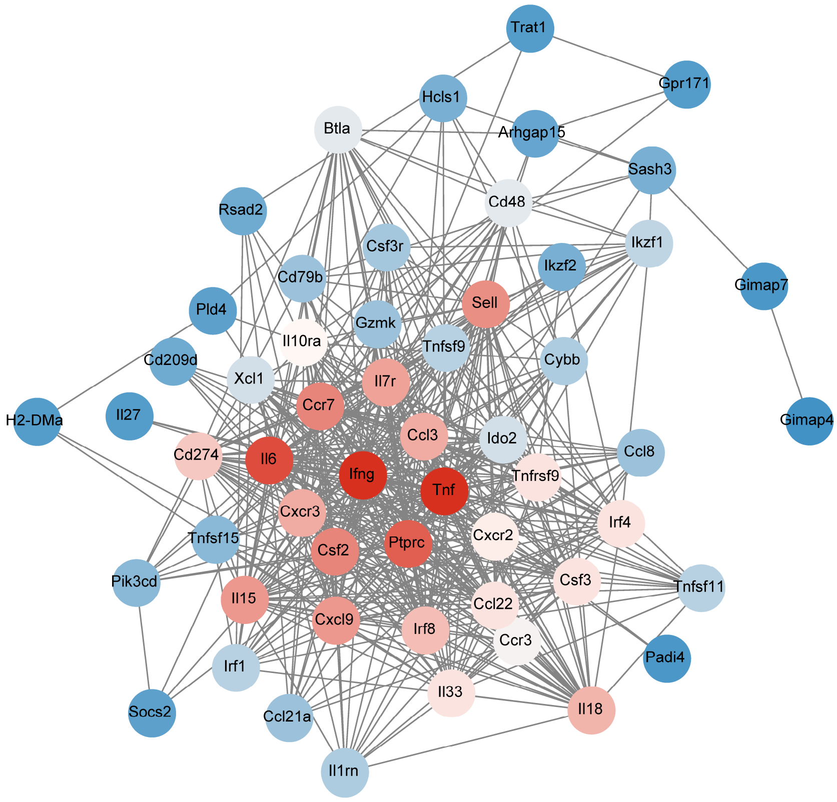

Supplementary Fig. 22. Protein-protein interaction (PPI) network of immune-related differentially expressed genes. The raw sequencing data have been deposited in the NCBI Sequence Read Archive (SRA) under the BioProject accession number PRJNA1288257. The data are publicly available and can be accessed through the NCBI SRA database.

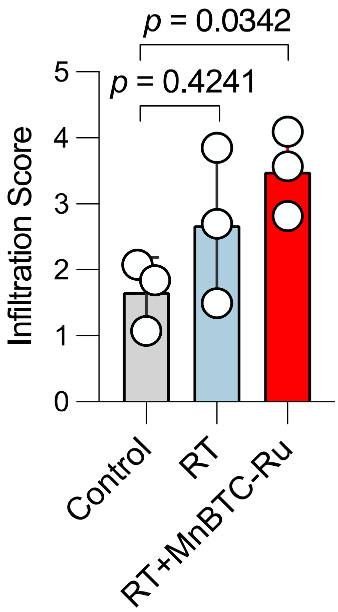

Supplementary Fig. 23. The immune infiltration score estimated based on gene expression profiles, reflects the overall level of immune activity, with higher scores indicating stronger immune cell infiltration (n = 3 independent experiments, data are presented as mean ± SD). Statistical significance was assessed using the one-way ANOVA for multiple-group comparisons, followed by Tukey's two-tailed post-hoc test for pairwise analysis, all tests were two-sided. Source data are provided as a Source Data file.

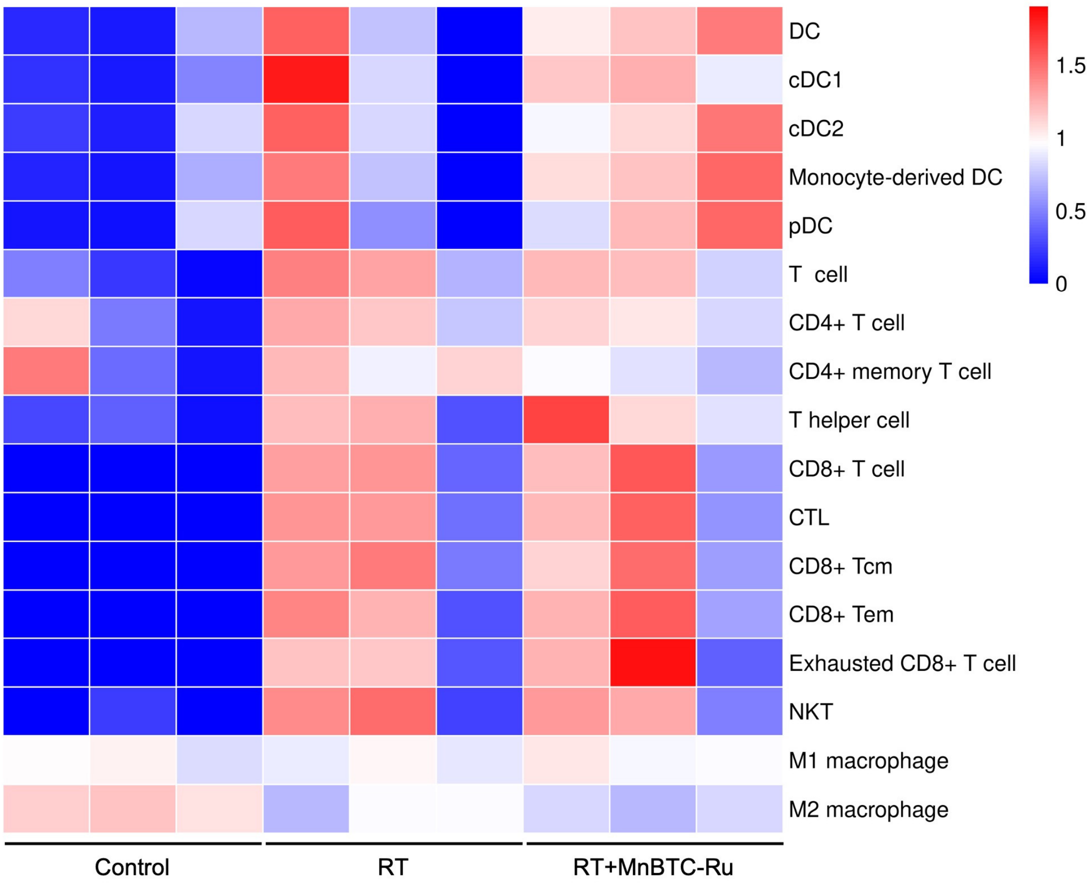

Supplementary Fig. 24. Heatmap showing the relative abundance of immune cell types deconvoluted from RNA-seq data. The intensity of the color gradient reflects the relative abundance of immune cells. DC indicates dendritic cell, CTL indicates Cytotoxic T Lymphocyte, NKT indicates Natural Killer T cell. The raw sequencing data have been deposited in the NCBI Sequence Read Archive (SRA) under the BioProject accession number PRJNA1288257. The data are publicly available and can be accessed through the NCBI SRA database.

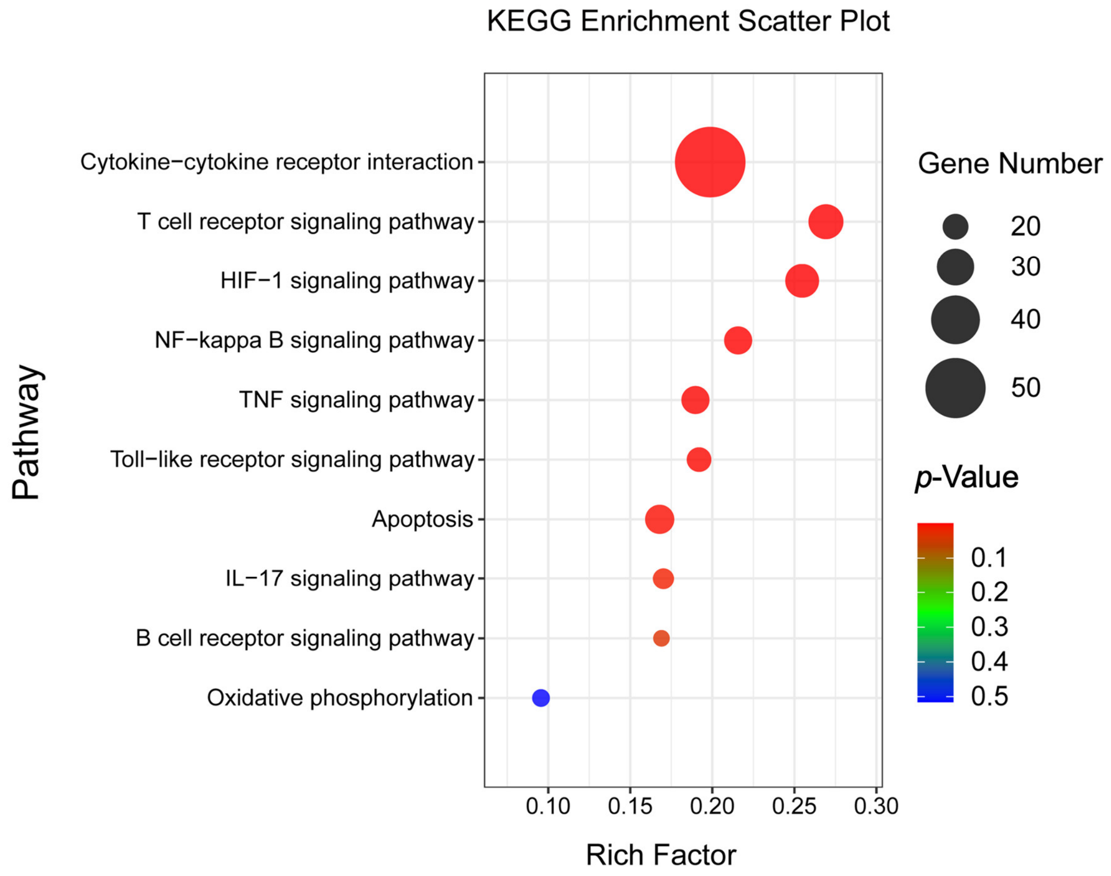

Supplementary Fig. 25. Kyoto Encyclopedia of Genes and Genomes (KEGG) enrichment analysis of upregulated genes in the RT+MnBTC-Ru group. HIF-1 indicates Hypoxia-Inducible Factor-1, NF-kappa B indicates Nuclear Factor Kappa-Light-Chain-Enhancer of Activated B Cells, TNF indicates Tumor Necrosis Factor, IL-17 indicates Interleukin-17. The raw sequencing data have been deposited in the NCBI Sequence Read Archive (SRA) under the BioProject accession number PRJNA1288257. The data are publicly available and can be accessed through the NCBI SRA database.

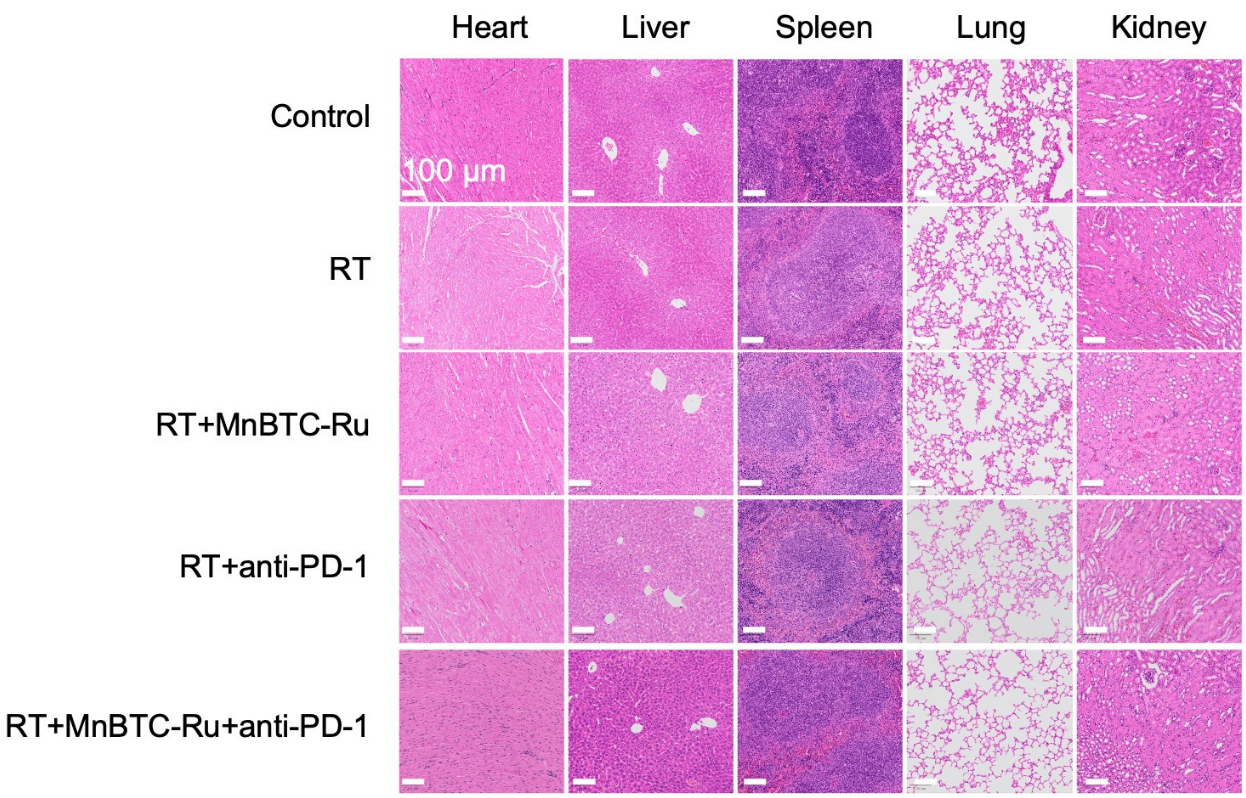

Supplementary Fig. 26. Representative H&E staining of major organs from CT26 tumor-bearing mice after different treatments (scale bar = 100 μm), experiments were repeated independently three times with similar results.

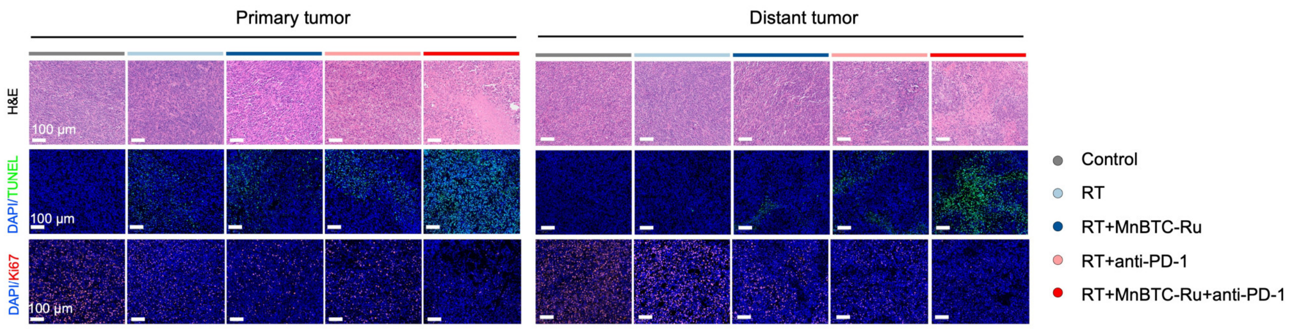

Supplementary Fig. 27. Representative H&E staining and immunofluorescence images of terminal deoxynucleotidyl transferase dUTP nick end labeling (TUNEL) assay and Ki67 expression from tumor

tissue sections slices (scale bar = 100 μm), experiments were repeated independently three times with similar results.

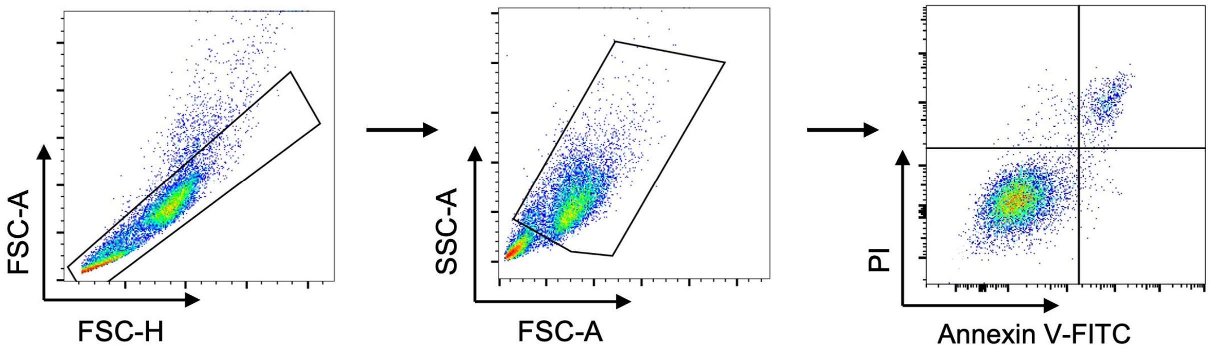

Supplementary Fig. 28. Gating strategy to determine the percentage of early apoptosis cells and late apoptosis cells, as displayed in Fig. 4g and Supplementary Fig. 15. FSC-A indicates Forward Scatter-Area, FSC-H indicates Forward Scatter-Height, SSC-A indicates Side Scatter-Area.

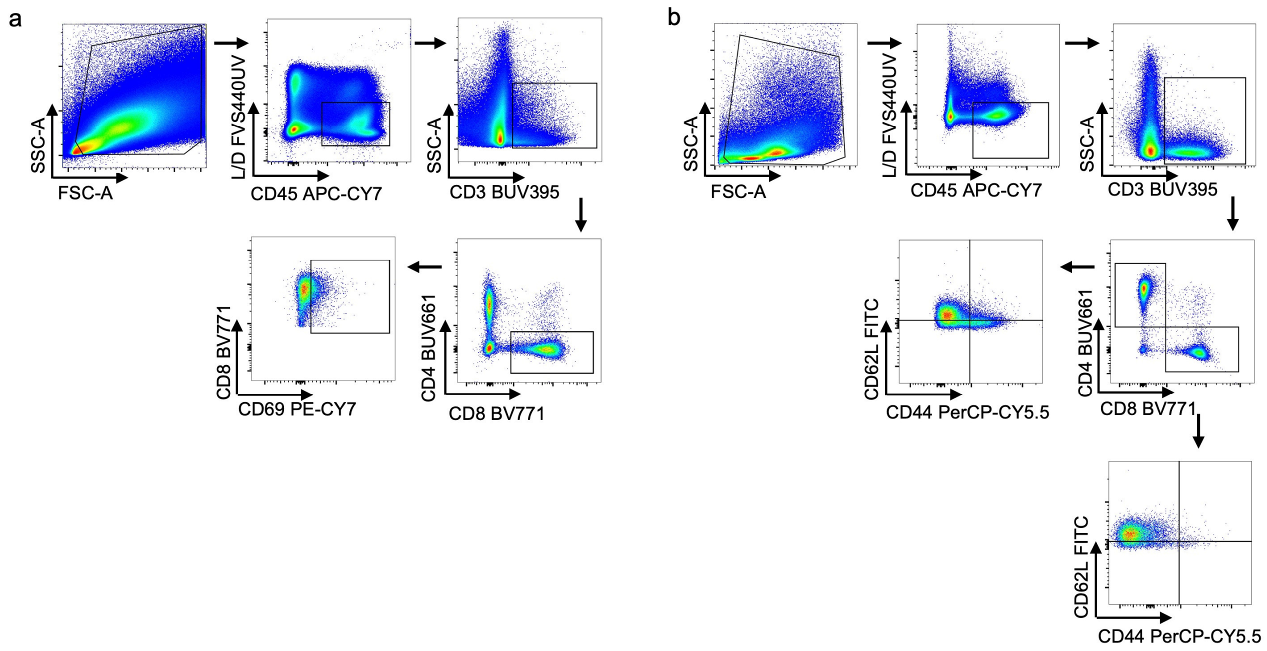

Supplementary Fig. 29. a Gating strategy to determine the percentage of T cells, CD8$^+$ T cells and CD8$^+$CD69$^+$ T cells, as displayed in Figure 5i. **b** Gating strategy to determine the percentage of T cells, CD4$^+$ T cells, CD8$^+$ T cells, CD4$^+$ central memory T cells (Tcms), CD4$^+$ effector memory T cells (Tems), CD8$^+$ Tcms and CD8$^+$ Tems, as displayed in Fig. 7k-o. L/D indicates Live/Dead.

Supplementary Tables

Supplementary Table 1. The elemental contents in the biocatalysts are determined by X-ray photoelectron spectroscopy (XPS) measurements.

XPS	Atomic (%)				Weight (%)			
	C	O	Mn	Ru	C	O	Mn	Ru
MnBTC-Ru	62.52	30.04	6.38	1.06	44.45	28.45	20.75	6.34
C-Ru	99.62	-	-	0.38	96.89	-	-	3.11

Supplementary Table 2. Comparison of the kinetics of MnBTC-Ru and C-Ru.

	E_0 (μM)	V_{max} (μM s^{-1})	K_m (mM)	TON (×10^{-3} s^{-1})
MnBTC-Ru	31.38	2.25	1.46	71.60
C-Ru	15.39	0.44	0.30	28.91

Compared to C-Ru, the MnBTC-Ru shows much higher TON, indicating more efficient catalytic kinetics.

Supplementary Table 3. Comparison of V_{max} and TON with recently reported state-of-the-art ROS-generation biocatalysts. TON=V_{max}/[E_0], where [E_0] is the mole concentration of metal in materials.

Artificial enzymes	$1/K_m$ (mM^{-1})	V_{max} (μM s^{-1})	TON (10^{-3} s^{-1})	Ref.
MnBTC-Ru	0.68	2.25	71.60	This work
CoO	1.09×10^{-2}	1.14	8.55	[1]
CeO_2	0.23	0.18	3.10	[1]
Mn_2O_3	7.98×10^{-2}	1.01	7.98	[1]
Fe_3O_4	2.40×10^{-2}	0.16	1.24	[1]
CuO	3.21×10^{-2}	0.28	2.24	[1]
Ru NPs	0.45	0.58	5.86	[2]
Pt nano-dendrites	0.14	9.9×10^{-2}	14.14	[3]
PtFe	4.56×10^{-3}	8.2×10^{-2}	2.20	[4]

Fe-MOF	769.23	2.5×10^{-2}	0.51	[5]
Fe-N-C	83.33	0.22	36.74	[6]
Zn-N-C single-atom enzyme	2.49×10^{-2}	0.12	2.53	[6]
Fe_2O_3	1.32×10^{-2}	6.8×10^{-2}	0.54	[1]
Co-N-C single-atom enzyme	6.15×10^{-2}	0.17	1.06	[7]
Co_3O_4	2.40×10^{-2}	0.26	2.09	[1]
Cu nanoparticle/N-C	5.56×10^{-2}	8.57×10^{-2}	3.32	[6]
$Cu(OH)_2$	3.46×10^{-2}	0.34	3.33	[1]
Fe-N-C single-atom enzyme	0.23	0.62	3.98	[7]
$PtFe@Fe_3O_4$	1.86×10^{-2}	0.11	8.62	[4]
Pd@Pt nano-dendrites	7.14×10^{-2}	0.09	12.86	[8]

Supplementary Methods

Characterizations.

Field emission SEM was performed with the Hitachi Regulus8220, Japan. The elemental mapping is obtained from an energy-dispersive X-ray detector (Quantax FlatQUAD, Bruker). The TEM, atomic-resolution HAADF-STEM, and EDS mapping were performed via a Talos F200x TEM microscope (FEI Ltd., USA) operated at 200 kV and analyzed by GMS-free analysis. X-ray diffraction (Ultima IV, Rigaku, Japan) was used to analyze the crystal structures of the catalysts, employing Cu Kα radiation over a 2θ range of 5-30°, with a scanning speed of 3°/min and a quartz sample stage. DLS was performed with Malvern Nano-ZS. XPS spectra were measured on the K-Alpha^{TM}+X-ray Photoelectron Spectrometer System (Thermo Scientific) with a Hemispheric 180° dual-focus analyzer with a 128-channel detector. A Nicolet-ls50 spectrophotometer (Nicol, US) was used for Fourier transform infrared (FTIR) spectroscopy analysis of the MnBTC and MnBTC-Ru in the range of 4000-500 cm^{-1} with a resolution of 2 cm^{-1}. In-situ FTIR spectroscopic analysis was performed using an infrared spectrometer (Thermo Scientific, iS50 FTIR) equipped with an insitu spectrum cell (Shanghai Yuanfang Technology Co., Ltd., SPECEL-III). The X-

ray absorption (XAS) spectra of Ru *K*-edge were conducted in fluorescence mode at the BL14W1 beamline of the Shanghai Synchrotron Radiation Facility, China, operated at 3.5 GeV with maximum injection currents of 230 mA. The synchrotron beam was monochromatized using a double-crystal monochromator equipped with a Si (111) crystal to reduce the harmonic component of the monochrome beam. The absorbance was measured using a multifunctional enzyme labeler (ReadMax 1900). Data analysis was performed with various software, including MDI Jade 6, Digital Micrograph 3.7.4, Avantage 5.967, Artemis software 0.9.26, Athena software 0.9.26, VASP 5.4.1, Origin 2024, and GraphPad Prism 10.2.3. DICOM images were processed and reconstructed into 3D models using Imaris software (10.2). Flow cytometry analysis was conducted using FlowJo v10.8.1, while bioinformatics analyses were performed on the free online platform (https://www.omicstudio.cn). All original schematic diagrams were created using the open-source software Blender 3.6 and Inkscape 1.4.2, both distributed under the GNU General Public License (GPL).

Density functional theory (DFT) calculation.

All theoretical calculations were performed using the DFT method, as implemented in the Vienna ab initio simulation package (VASP). The core electrons were described using the spin-polarized projector augmented wave (PAW) method, and the electron exchange and correlation energy were treated within the generalized gradient approximation in the Perdew-Burke-Ernzerhof functional (GGA-PBE). The valence states of all atoms were expanded in a plane-wave basis set with a cutoff energy of 450 eV. The convergence criteria for the electronic self-consistent iteration and force were set to 10^{-5} eV and 0.05 eV/Å with a Gamma centered 2×1×1 K-points. Denser 4×2×1 K-points were used for the density of states (DOS) computations. The slab model was constructed with a vacuum layer of 16 Å in the z-direction to avoid the interaction between neighboring images. The charge density differences were evaluated using the formula (1):

$$\Delta\rho = \rho_{A+B} - \rho_A - \rho_B \quad (1)$$

where ρ_X is the electron density of X. Atomic charges were computed using the atom-in-molecule (AIM) scheme proposed by Bader. The isosurface value used for differential charge density is 0.0045 e $Å^{-3}$.

To quantitatively describe the adsorption ability of loading materials, the adsorption strength is defined as Equation (2):

$$E_{ads} = E_{adsorbate/substrate} - E_{adsorbate} - E_{substrate} \quad (2)$$

where $E_{adsorbate/substrate}$, $E_{substrate}$, and $E_{adsorbate}$ represent the total energy of the substrate with adsorbed species, the clean substrate, and the molecule in the gas phase, respectively.

Details for in-situ FTIR measurement.

We typically prepare a Nafion solution consisting of 210 μL of isopropyl alcohol, 750 μL of deionized water, and 40 μL of Nafion (Perfluorosulfonic acid ion exchange resin, Energy Chemical, 5% w/w in 1-propanol and water). Subsequently, a catalyst/Nafion dispersion was formulated to achieve a final concentration of 10 mg/mL. During the experiment, we deposited 40 μL of the catalyst solution onto a ZnSe crystal surface, allowed it to dry, and then installed the crystal into the in-situ cell. Subsequently, 5 mL of NaOAc−HOAc buffer (100 mM, pH 4.5) containing H_2O_2 (0.5 M) was included in the in-situ cell, and we collected the in-situ FTIR spectra at specific intervals, maintaining a reaction time of 10 min.

RNA-seq and bioinformatics analysis.

Tumor samples were collected for RNA sequencing, total RNA was extracted from tumor tissues using TROzol Reagent (Thermo Fisher, 15596018). RNA libraries were constructed using Bioanalyzer 2100 and RNA 6000 Nano LabChip Kit (Agilent, CA, USA) following manufacturer' s instructions, ensuring the creation of high-quality RNA sequencing libraries. These libraries were then sequenced using the Illumina Novaseq 6000™ platform (LC-Bio Technology CO., Ltd., Hangzhou, China), generating double-ended reads for subsequent bioinformatics analysis.

Figures and artwork.

Graphic elements in Figs. 1a, 1b, 2i, 3a, 3m, 4a, 4j, 5a, 7a, 7h, and Supplementary Fig. 7 were created using the opensource software Blender 3.6 and Inkscape 1.4.2, both distributed under the GNU General Public License (GPL).

Supplementary References

1 Wang, X. *et al.* eg occupancy as an effective descriptor for the catalytic activity of perovskite oxide-based peroxidase mimics. *Nat. Commun.* **10**, 704 (2019).

2 Cao, G.-J., Jiang, X., Zhang, H., Croley, T. R. & Yin, J.-J. Mimicking horseradish peroxidase and oxidase using ruthenium nanomaterials. *RSC Adv.* **7**, 52210-52217 (2017).

3 Ge, C. *et al.* Synthesis of Pt Hollow Nanodendrites with Enhanced Peroxidase-Like Activity against Bacterial Infections: Implication for Wound Healing. *Adv. Funct. Mater.* **28**, 1801484 (2018).

4 Li, S. *et al.* A Nanozyme with Photo-Enhanced Dual Enzyme-Like Activities for Deep Pancreatic Cancer Therapy. *Angew. Chem. Int. Ed.* **58**, 12624-12631 (2019).

5 Xu, W. *et al.* Glucose Oxidase-Integrated Metal-Organic Framework Hybrids as Biomimetic Cascade Nanozymes for Ultrasensitive Glucose Biosensing. *ACS Appl. Mater. Interfaces* **11**, 22096-22101 (2019).

6 Wu, Y. *et al.* Cascade Reaction System Integrating Single-Atom Nanozymes with Abundant Cu Sites for Enhanced Biosensing. *Anal. Chem.* **92**, 3373-3379 (2020).

7 Jiao, L. *et al.* Densely Isolated FeN_4 Sites for Peroxidase Mimicking. *ACS Catal.* **10**, 6422-6429 (2020).

8 Wu, R. *et al.* Synthesis of Pt Hollow Nanodendrites with Enhanced Peroxidase-Like Activity against Bacterial Infections: Implication for Wound Healing. *Adv. Funct. Mater.* **28**, 1801484 (2018).
