# Supplementary material for: Bioinspired ruthenium-manganese-oxygen complex for biocatalytic and radiosensitization therapies to eradicate primary and metastatic tumors
Source: Nat Commun. 2025 Aug 16;16:7640. doi: 10.1038/s41467-025-62999-x (PMC12357870; doi:10.1038/s41467-025-62999-x)
Supplement: Supplementary file 2 — Reporting Summary [file 41467_2025_62999_MOESM2_ESM.pdf]

nature portfolio

Corresponding author(s): Chong Cheng

Last updated by author(s): Jul 9, 2025

Reporting Summary

Nature Portfolio wishes to improve the reproducibility of the work that we publish. This form provides structure for consistency and transparency in reporting. For further information on Nature Portfolio policies, see our Editorial Policies and the Editorial Policy Checklist.

Statistics

For all statistical analyses, confirm that the following items are present in the figure legend, table legend, main text, or Methods section.

n/a	Confirmed	
☐	☒	The exact sample size (n) for each experimental group/condition, given as a discrete number and unit of measurement
☐	☒	A statement on whether measurements were taken from distinct samples or whether the same sample was measured repeatedly
☐	☒	The statistical test(s) used AND whether they are one- or two-sided<br>*Only common tests should be described solely by name; describe more complex techniques in the Methods section.*
☒	☐	A description of all covariates tested
☒	☐	A description of any assumptions or corrections, such as tests of normality and adjustment for multiple comparisons
☐	☒	A full description of the statistical parameters including central tendency (e.g. means) or other basic estimates (e.g. regression coefficient) AND variation (e.g. standard deviation) or associated estimates of uncertainty (e.g. confidence intervals)
☐	☒	For null hypothesis testing, the test statistic (e.g. F, t, r) with confidence intervals, effect sizes, degrees of freedom and P value noted<br>*Give P values as exact values whenever suitable.*
☒	☐	For Bayesian analysis, information on the choice of priors and Markov chain Monte Carlo settings
☒	☐	For hierarchical and complex designs, identification of the appropriate level for tests and full reporting of outcomes
☒	☐	Estimates of effect sizes (e.g. Cohen's d, Pearson's r), indicating how they were calculated

Our web collection on statistics for biologists contains articles on many of the points above.

Software and code

Policy information about availability of computer code

Data collection

Field emission scanning electron microscopy (FE-SEM) was performed with the Hitachi Regulus8220, Japan. The elemental mapping is obtained from an energy-dispersive X-ray detector (Quantax FlatQUAD, Bruker). The transmission electron microscopy (TEM), atomic-resolution high-angle annular dark-field scanning TEM, and energy dispersive spectroscopy (EDS) mapping were performed via a Talos F200x TEM microscope (FEI Ltd., USA) operated at 200 kV and analyzed by GMS-free analysis. X-ray diffraction (Ultima IV, Rigaku, Japan) was used to analyze the crystal structures of the catalysts, employing Cu Kα radiation over a 2θ range of 5-30°, with a scanning speed of 3°/min and a quartz sample stage. Dynamic light scattering (DLS) was performed with Malvern Nano-ZS. X-ray photoelectron spectroscopy (XPS) spectra were measured on the K-Alpha™ + X-ray Photoelectron Spectrometer System (Thermo Scientific) with a Hemispheric 180° dual-focus analyzer with a 128-channel detector. A Nicolet-Is50 spectrophotometer (Nicol, US) was used for Fourier transform infrared (FTIR) spectroscopy analysis of the MnBTC and MnBTC-Ru in the range of 4000-500 cm-1 with a resolution of 2 cm-1. In-situ FTIR spectroscopic analysis was performed using an infrared spectrometer (Thermo Scientific, iS50 FTIR) equipped with an insitu spectrum cell (Shanghai Yuanfang Technology Co., Ltd., SPECEL-III). The X-ray absorption (XAS) spectra of Ru K-edge were conducted in fluorescence mode at the BL14W1 beamline of the Shanghai Synchrotron Radiation Facility, China, operated at 3.5 GeV with maximum injection currents of 230 mA. The synchrotron beam was monochromatized using a double-crystal monochromator equipped with a Si (111) crystal to reduce the harmonic component of the monochrome beam. The absorbance was measured using a multifunctional enzyme labeler (ReadMax 1900). Fluorescence images were collected via by the Olympus IX83 Live Microscope (Olympus Life Science, Japan). In vitro Flow-cytometry data were collected via a flow cytometer (CytoFLEX, Beckman, USA). RNA libraries were constructed using Bioanalyzer 2100 and RNA 6000 Nano LabChip Kit (Agilent, CA, USA) following manufacturer' s instructions. These libraries were then sequenced using the Illumina Novaseq 6000TM platform (LC-Bio Technology CO., Ltd., Hangzhou, China). Multicolor immunoflow cytometry stained cells were determined using BD FACS FCM (FACSymphony A5, BD, USA) . FlowJo 10.8.1 was used for flow cytometry data analysis. In vivo H&E staining and fluorescence imaging were scanned by a full slide scanner (VS200, Olympus, Japan). Micro-CT images were collected using CT Scanner (NEMO micro-CT, Pingseng Scientific, China). The Ru content in tissue was quantified using inductively coupled plasma mass spectrometry (ICP-MS, Agilent 7850) analysis.

Data analysis

Data analysis was performed with various software, including MDI Jade 6, Digital Micrograph 3.7.4, Avantage 5.967, Artemis software 0.9.26, Athena software 0.9.26, VASP 5.4.1, Origin 2024, and GraphPad Prism 10.2.3. DICOM images were processed and reconstructed into 3D models using Imaris software (10.2). Flow cytometry analysis was conducted using FlowJo v10.8.1, while bioinformatics analyses were performed on the free online platform (https://www.omicstudio.cn). All original schematic diagrams were created using the open-source software Blender 3.6 and Inkscape 1.4.2, both distributed under the GNU General Public License (GPL).

For manuscripts utilizing custom algorithms or software that are central to the research but not yet described in published literature, software must be made available to editors and reviewers. We strongly encourage code deposition in a community repository (e.g. GitHub). See the Nature Portfolio guidelines for submitting code & software for further information.

Data

Policy information about availability of data

All manuscripts must include a data availability statement. This statement should provide the following information, where applicable:

- Accession codes, unique identifiers, or web links for publicly available datasets
- A description of any restrictions on data availability
- For clinical datasets or third party data, please ensure that the statement adheres to our policy

All data supporting the results of this study are available within the paper and its Supplementary Information. All raw data generated for the figures in this study are provided in the source data file. Source data are available for Figs. 1c, 1g, 1k, 2a, 2b, 2c, 2d, 2e, 2f, 2g, 2h, 3b, 3c, 3d, 3e, 3f, 3g, 3h, 3i, 3j, 3k, 3l, 3n, 3o, 4d, 4e, 4g, 4i, 4k, 5b, 5c, 5d, 5e, 5h, 5i, 6a, 7b, 7c, 7d, 7e, 7f, 7i, 7j, 7k, 7l, 7m, 7n, and 7o, and Supplementary Figs. 1, 4, 8, 9, 10, 11, 12, 13, 15, 16, 17, 19, 20, 21, and 23 in the associated source data file. The raw sequencing data generated in this study have been deposited in the NCBI Sequence Read Archive (SRA) under the BioProject accession number PRJNA1288257. These data are publicly available and can be accessed through the NCBI SRA database. Source data are provided with this paper.

Research involving human participants, their data, or biological material

Policy information about studies with human participants or human data. See also policy information about sex, gender (identity/presentation), and sexual orientation and race, ethnicity and racism.

Reporting on sex and gender	N/A
Reporting on race, ethnicity, or other socially relevant groupings	N/A
Population characteristics	N/A
Recruitment	N/A
Ethics oversight	N/A

Note that full information on the approval of the study protocol must also be provided in the manuscript.

Field-specific reporting

Please select the one below that is the best fit for your research. If you are not sure, read the appropriate sections before making your selection.

☒ Life sciences ☐ Behavioural & social sciences ☐ Ecological, evolutionary & environmental sciences

For a reference copy of the document with all sections, see nature.com/documents/nr-reporting-summary-flat.pdf

Life sciences study design

All studies must disclose on these points even when the disclosure is negative.

Sample size	For all in vitro studies, at least three random samples were selected foe statistical analysis in each experiment based on the effect size and overlap between distributions. For all in vivo studies, at least three random samples were selected for statistical analysis in each experiment. The sample sizes for all experiments were determined based on prior studies, aiming to ensure statistical power while minimizing resource use. No formal sample size calculation was performed; instead, sample sizes were chosen to provide sufficient reliability based on previous literature and experimental design. (Cell Metab. 2024, 36, 2493-2510e9; Cancer Cell. 2023, 41, 272-287.e9).
Data exclusions	No data was excluded from the analysis.
Replication	Results shown in the manuscript are representative of at least three independent experiments. All our attempts at replication were successful with similar results.
Randomization	Our samples/organisms were allocated randomly.
Blinding	In all experiments, investigators were blinded to group allocation during data collection and processing.

Reporting for specific materials, systems and methods

We require information from authors about some types of materials, experimental systems and methods used in many studies. Here, indicate whether each material, system or method listed is relevant to your study. If you are not sure if a list item applies to your research, read the appropriate section before selecting a response.

Materials & experimental systems

n/a	Involved in the study
☐	☒ Antibodies
☐	☒ Eukaryotic cell lines
☒	☐ Palaeontology and archaeology
☐	☒ Animals and other organisms
☒	☐ Clinical data
☒	☐ Dual use research of concern
☒	☐ Plants

Methods

n/a	Involved in the study
☒	☐ ChIP-seq
☐	☒ Flow cytometry
☒	☐ MRI-based neuroimaging

Antibodies

Antibodies used	In vitro analysis: anti-HIF-1α antibody (Abcam, Catalog No. ab179483, 1:500 dilution), anti-CRT antibody (Abcam, Catalog No. ab92516, 1:500 dilution).<br>In vivo fluorescence imaging: anti-γ-H2AX antibody (Servicebio, Catalog No. GB111841, 1:100 dilution), anti-Ki67 antibody (Servicebio, Catalog No. GB111141, 1:500 dilution) and anti-CD8 antibody (Servicebio, Catalog No. GB15068, 1:1000 dilution).<br>FCM analysis: FVS440UV (BD, Catalog No. 566332, 1:1000 dilution), purified Rat Anti-Mouse CD16/CD32 (BD, Catalog No. 553141, 0.5 µg per test), anti-CD45-APC-CY7 (BD, Catalog No. 557659), anti-CD3-BUV395 (BD, Catalog No. 563565), anti-CD4-BUV661 (BD, Catalog No. 741461), anti-CD8-BV771 (BD, Catalog No. 563046), anti-CD69-PE-CY7 (BD, Catalog No. 552879), anti-CD44-PerCP-Cy5.5 (BD, Catalog No. 560570), anti-CD62L-FITC (BD, Catalog No. 561917). All antibodies were diluted to the working concentration of 0.2 µg per test.
Validation	All antibodies were verified by the supplier and each lot has been quality tested. All validation statements of primary antibodies can be found on the respective antibody website:<br>anti-HIF-1α: https://www.abcam.cn/products/primary-antibodies/hif-1-alpha-antibody-epr16897-ab179483.html<br>anti-CRT: https://www.abcam.cn/products/primary-antibodies/calreticulin-antibody-epr3924-er-marker-ab92516.html<br>anti-γ-H2AX: https://www.servicebio.cn/goodsdetail?id=4702<br>anti-Ki67: https://www.servicebio.cn/goodsdetail?id=2828<br>anti-CD8: https://www.servicebio.cn/goodsdetail?id=13908<br>FVS440UV: https://www.bdbiosciences.com/en-at/products/reagents/flow-cytometry-reagents/research-reagents/single-color-antibodies-ruo/fixable-viability-stain-440uv.566332?tab=product_details<br>purified Rat Anti-Mouse CD16/CD32: https://www.bdbiosciences.com/en-at/products/reagents/western-blotting-and-molecular-reagents/purified-rat-anti-mouse-cd16-cd32-mouse-bd-fc-block.553141?tab=product_details<br>anti-CD45-APC-CY7: https://www.bdbiosciences.com/en-at/products/reagents/flow-cytometry-reagents/research-reagents/single-color-antibodies-ruo/apc-cy-7-rat-anti-mouse-cd45.557659?tab=product_details<br>anti-CD3-BUV395: https://www.bdbiosciences.com/en-at/products/reagents/flow-cytometry-reagents/research-reagents/single-color-antibodies-ruo/buv395-hamster-anti-mouse-cd3e.563565?tab=product_details<br>anti-CD4-BUV661: https://www.bdbiosciences.com/en-at/products/reagents/flow-cytometry-reagents/research-reagents/single-color-antibodies-ruo/buv661-rat-anti-mouse-cd4.569752?tab=product_details<br>anti-CD8-BV771: https://www.bdbiosciences.com/en-at/products/reagents/flow-cytometry-reagents/research-reagents/single-color-antibodies-ruo/bv711-rat-anti-mouse-cd8a.563046?tab=product_details<br>anti-CD69-PE-CY7: https://www.bdbiosciences.com/en-at/products/reagents/flow-cytometry-reagents/research-reagents/single-color-antibodies-ruo/pe-cy-7-hamster-anti-mouse-cd69.552879?tab=product_details<br>anti-CD44-PerCP-Cy5.5: https://www.bdbiosciences.com/en-at/products/reagents/flow-cytometry-reagents/research-reagents/single-color-antibodies-ruo/percp-cy-5-5-rat-anti-mouse-cd44.560570?tab=product_details<br>anti-CD62L-FITC: https://www.bdbiosciences.com/en-at/products/reagents/flow-cytometry-reagents/research-reagents/single-color-antibodies-ruo/fitc-rat-anti-mouse-cd62l.561917?tab=product_details

Eukaryotic cell lines

Policy information about cell lines and Sex and Gender in Research

Cell line source(s)	CT26
Authentication	The cell line was authenticated.
Mycoplasma contamination	Cell line were not contaminated by Mycoplasma.
Commonly misidentified lines<br>(See ICLAC register)	No commonly misidentified cell line was used.

Animals and other research organisms

Policy information about studies involving animals; ARRIVE guidelines recommended for reporting animal research, and Sex and Gender in Research

Laboratory animals	Female BALB/c mice (6–8 weeks old) were purchased from Jiangsu Gempharmatech Co., Ltd. and housed in a specific pathogen-free facility. All mice were maintained under controlled conditions with a 12-hour light/dark cycle (lights on from 8:00 a.m. to 8:00 p.m.), constant temperature (18–22°C), and humidity (50–60%), with unrestricted access to standard chow and water.
Wild animals	The study did not involve wild animals.
Reporting on sex	Female mice were selected in this study. n = 5 biologically independent mice per group.
Field-collected samples	The study did not involve samples collected from the field.
Ethics oversight	The animal experiments were approved by the Institutional Animal Care and Use Committee of West China Hospital, Sichuan University (Approval No. 20241017001). All animal experiments were conducted in accordance with the ARRIVE guidelines.

Note that full information on the approval of the study protocol must also be provided in the manuscript.

Plants

Seed stocks	N/A
Novel plant genotypes	N/A
Authentication	N/A

Flow Cytometry

Plots

Confirm that:

☒ The axis labels state the marker and fluorochrome used (e.g. CD4-FITC).

☒ The axis scales are clearly visible. Include numbers along axes only for bottom left plot of group (a 'group' is an analysis of identical markers).

☒ All plots are contour plots with outliers or pseudocolor plots.

☒ A numerical value for number of cells or percentage (with statistics) is provided.

Methodology

Sample preparation	1. Tumor : Euthanize the mouse following ethical guidelines and institutional protocols. Excise the tumor tissue immediately post-mortem to avoid cell degradation. Place the tumor in cold RPMI-1640 supplemented with 2% FBS (Fetal Bovine Serum) to maintain cell viability. Tumor dissociation can be performed by either enzymatic digestion or mechanical disaggregation. Typically, the combination of both provides optimal results. Prepare a digestion buffer such as: collagenase Type IV (1-2 mg/mL), DNase I (50 U/mL), and Hyaluronidase (1 mg/mL) in RPMI-1640 or PBS. Mince the tumor tissue into small pieces (1–3 mm^3) using sterile scissors or a scalpel in a Petri dish. Transfer the minced tissue into a 15 mL or 50 mL conical tube containing digestion buffer. Incubate the sample at 37°C for 30–60 minutes with gentle agitation on a rocker or shaker. Following enzymatic digestion, gently triturate the tissue using a wide-bore pipette to break apart clumps and release cells. Filter the dissociated cells through a 40 µm nylon cell strainer into a sterile 50 mL tube to remove clumps and undigested materials. Centrifuge the strained cell suspension at 300–400 x g for 5 minutes at 4°C. Remove the supernatant, resuspend the cell pellet in cold PBS or Flow Staining Buffer . Repeat washing 1–2 times to remove residual enzymes and debris.<br>2. Spleen: Carefully excise the spleen using sterile dissection tools. Place the excised spleen in cold complete RPMI-1640 medium supplemented with: 2% FBS (Fetal Bovine Serum). Keep the spleen on ice until further processing. Place the spleen in a 70 µm cell strainer over a 50 mL Falcon tube. Add 2–5 mL of RPMI-1640 with 2% FBS to the strainer. Gently press the spleen against the mesh using the plunger of a sterile 5 mL syringe or a rubber pestle. Perform circular motions to mechanically break down the spleen tissue and release cells. Wash the mesh with an additional 2–3 mL of cold PBS or RPMI to ensure maximum recovery of cells. Collect the filtered cell suspension and centrifuge at 300–400 x g for 5 minutes at 4°C.
Instrument	BD, FACSymphony A5

Software

Flow-cytometry analysis was performed in FlowJo v10.8.1.

Cell population abundance

At least 50,000 relevant events were acquired for in vivo flow cytometry analysis.

Gating strategy

The gating strategies were shown in Supplementary data. For in vivo flow cytometry analysis, live cells were gated as FVS440UV-negative populations , followed by selection of CD45+ cells to define the immune cell population . Among CD3+ T cells, subsets were further differentiated into CD4+ and CD8+ populations . Early activation status was analyzed based on CD69 expression, while effector memory T cells and central memory T cells were identified according to CD44/CD62L expression patterns.

☒ Tick this box to confirm that a figure exemplifying the gating strategy is provided in the Supplementary Information.
